# A community‐driven approach to address substance use and create a Great Plains American Indian addiction and recovery research agenda

**DOI:** 10.1002/ajcp.70039

**Published:** 2025-12-26

**Authors:** Brynn Luger, Anna Kihlström, Brinda Sivaramakrishnan, Allison Kelliher, Frankie Kropp, Carmen Rosa, T. John Winhusen, Donald Warne, the CTN0129 participating Tribal communities

**Affiliations:** ^1^ Department of Indigenous Health University of North Dakota School of Medicine & Health Sciences Grand Forks North Dakota USA; ^2^ Center for Indigenous Health, Bloomberg School of Public Health Johns Hopkins University Baltimore Maryland USA; ^3^ Center for Indigenous Health, Johns Hopkins Bloomberg School of Public Health, and School of Nursing Johns Hopkins University Baltimore Maryland USA; ^4^ Department of Psychiatry and Behavioral Neuroscience University of Cincinnati College of Medicine Cincinnati Ohio USA; ^5^ Center for the Clinical Trials Network National Institute on Drug Abuse, Bethesda, MD (retired); ^6^ Center for Addiction Research University of Cincinnati College of Medicine Cincinnati Ohio USA; ^7^ Center for Indigenous Health Johns Hopkins Bloomberg School of Public Health Baltimore Maryland USA

**Keywords:** American Indian, ceremony assisted treatment, community‐based participatory research, substance use

## Abstract

Substance use, specifically opioid and methamphetamine use, is of increasing concern among American Indian (AI) populations in the Great Plains. This community‐driven participatory study investigated the impacts of substance use and community‐defined needs in treating addiction. It determined the priorities for future research on behavioral health and substance misuse in the Great Plains region. Behavioral health and social services professionals and community stakeholders were identified from eight Great Plains communities and invited to attend eight focus groups (*N* = 47). Conversations were audio‐recorded, transcribed, and coded by the research team. The qualitative data analysis identified four themes: (1) Challenges with Treatment and Recovery, (2) Impact of Substance Use, (3) Reasons for Substance Use, (4) Solutions and Research Priorities. The findings highlight barriers to substance use disorder (SUD) treatment ranging from policy issues to lack of funding. The most significant finding centered on integrating cultural strengths into treatment and recovery programs, including Ceremony Assisted Treatment (CAT). Data reports for each participating organization were provided to disseminate outcomes in their respective communities. Other key findings suggest that addressing the root causes of substance use disorder, along with early intervention and comprehensive counseling services, are essential for long‐term success.

## Highlights

- Impacts of substance use in Great Plains American Indian Tribal communities.
- Engagement with Great Plains Tribal communities to guide future research priorities.
- Integrating cultural strengths into treatment and recovery programs is necessary.
- Critical issue of addiction and associated community challenges in the Great Plains Tribal region.

## INTRODUCTION

Due to the myriad impacts of colonization, substance misuse has been a long‐standing concern for many American Indian (AI) individuals and communities (Substance Abuse and Mental Health Services Administration, 2019; Young & Joe, 2009). Specifically, there is increasing concern about opioid and methamphetamine use among AI populations. Opioid misuse, overdose, and death rates have disproportionately affected the health of AI populations (Ahmad et al., 2021; Komro et al., 2023). Increasing reports on the impacts of Opioid Use Disorder (OUD) and methamphetamine use within AI communities have focused attention on efforts to address factors contributing to substance use, as well as its individual, family, legal, and community‐level consequences. Problems that the present study aimed to address include: (1) a dearth of experiential data from frontline AI behavioral health service professionals on current needs and gaps for culturally appropriate substance use recovery and (2) a research and practice agenda to address the epidemic of substance use disorder that is community‐driven and generated through Indigenous community scholarship.

Though extremely resilient, the impacts of government policies on American Indian/Alaska Native (AI/AN) communities, such as forced removals and land dispossessions, have contributed to intergenerational transmission of health disparities (Komro et al., 2023). Significant health disparities persist among AI/ANs, such as higher rates of death from diabetes, chronic liver disease, cardiovascular disease, substance use, and intentional self‐harm/suicide compared to those of the general American population Indian Health Service IHS, 2019; Leung et al., 2023). Disparities also exist within Tribal Nations. For example, AI Tribes in the Great Plains region (North Dakota, South Dakota, Nebraska, and Iowa) experience health and socioeconomic disparities at rates greater than other US Tribal regions (Christensen & Kightlinger, 2013; Indian Health Service, 2015; Kunitz et al., 2014).

The Great Plains region is home to several distinct Tribal nations, among them are the Lakota, Omaha, Ponca, Sac and Fox Tribes, and others. It includes 16 reservations located in rural areas as well as AI populations living in non‐reservation (urban) settings (Indian Health Service [IHS], 2024). Most AI reservations are located in rural areas with small populations stretching over large land areas. Treatment resources in these regions are geographically dispersed, adding to the long list of barriers to substance use disorder (SUD) treatment. These communities often face significant challenges due to health and other infrastructure gaps. Urban settings present their own set of difficulties, such as engaging with AI/AN people in prevention and treatment efforts (Komro et al., 2023). AI communities in the Great Plains are diverse, each with unique concerns and priorities for addressing addiction and supporting recovery based on local needs.

Previous studies with AI people in the Great Plains have helped identify epidemiologic information, disparities, attitudes, and barriers around SUD and SUD treatment. However, to our knowledge, none have approached Great Plains AI people to ask about their priorities for addressing these issues. Therefore, research grounded in collaborative partnerships between AI communities and research institutions is needed to determine AI research priorities in these areas. Tribal Participatory Research (TPR) synthesizes community‐based participatory research (CBPR) approaches with the protection of Tribal interests when performing research with AI communities (Fisher & Ball, 2002, 2003, 2005). In addition to fostering collaboration and emphasizing the inclusion of community voices (Wallerstein et al., 2018), TPR approaches methodological issues to address health, social, and economic disparities within the framework of historical trauma. The study utilized a TPR approach to empower communities to speak for themselves and to improve the research′s overall fit and quality.

### Positionality

Our author team consists of eight researchers from various backgrounds, including three who identify as AI/AN (two Lakota Tribal members and one Koyukon Athabascan, Dena), one person of Tamil heritage, three of European descent, and one who identifies as Puerto Rican. This multidisciplinary team combines knowledge from public health, family medicine, integrative medicine, clinical psychology, and traditional Indigenous healing, as well as perspectives from two doctoral students in the discipline of Indigenous Health. Furthermore, our team bridges two centers renowned for research relating to AI/AN peoples: the University of North Dakota School of Medicine & Health Sciences Department of Indigenous Health and the Johns Hopkins University Center for Indigenous Health.

## MATERIALS AND METHODS

A qualitative approach, guided by a TPR framework, was used to explore community‐identified substance use issues and potential research priorities in the Great Plains tribal region. In addition to obtaining approvals from Tribe‐specific regulatory bodies, ongoing tribal oversight was provided by members of the Great Plains Behavioral Health Directors Association (GPBHDA) during study development, throughout the implementation phase, and during the dissemination of results to participating sites and Tribal communities. The GPBHDA convenes regularly to discuss and develop solutions to challenges facing treatment programs serving members of the Great Plains Tribal Nations. As needed, members of the GPBHDA served as facilitators in meetings with Tribal regulatory bodies to ensure both the needs of the study and the needs of the community were addressed. Project staff included members of two Great Plains Tribes, as well as graduate‐level students who were being trained specifically in addressing health concerns with AI communities. Following data analysis, participating communities were engaged to provide feedback on the findings before finalization. Traditional AI culture informed the format of data‐gathering sessions with the inclusion of prayer, refreshments, and so forth as appropriate.

### Procedures

The study aimed to engage both urban and reservation‐based substance use treatment programs. These programs differed in the services they offered, providing SUD treatment to adult and adolescent AIs. Services included assessments, detoxification, outpatient individual and group counseling, residential treatment, wrap‐around services, and, in some cases, culturally specific ceremonies such as sweat lodge purification ceremonies and Traditional naming ceremonies.

The research team secured approval from the University of North Dakota Institutional Review Board (IRB) for the overall study and obtained necessary approvals from Tribal IRBs or other Tribal regulatory bodies before recruiting participants from sites under Tribal jurisdiction. The IRB also granted a waiver of written consent for this minimal‐risk study.

After obtaining approvals, the study team engaged the GPBHD to assist in recruiting AI behavioral health service providers, professionals, and Tribal community stakeholders using a snowball sampling plan. Interested participants reviewed an IRB‐approved Study Information Sheet, which served as the study Informed Consent Document, and discussed any questions about the study before agreeing to participate. Focus groups occurred in‐person at the participating treatment programs. The study team provided a $25 retail voucher as compensation for completing the focus group.

Following analysis of the collected data, investigators prepared and shared reports of site‐ or Tribe‐specific findings to the respective participating urban and reservation‐based programs and their associated Tribal leadership, eliciting feedback and discussion of the results. In line with the principles of Indigenous data sovereignty, every site was encouraged to disseminate the study results as they deemed appropriate. Additionally, investigators shared a deidentified, aggregated data report with each participating site and with the GPBHDA, providing an overview of the Great Plains tribal area research priorities. Investigators presented the results in person to facilitate discussion and engagement. From this, investigators prepared the final research agenda, which was presented to the GPBHDA, participating sites, and their affiliated Tribal authorities.

### Measures

The study team developed an interview guide for focus groups in collaboration with community stakeholders; this guide was approved by the University of North Dakota IRB. Questions included specific substances misused in the community, barriers to treatment, prevention strategies, the role of historical and other forms of trauma in substance use, mistrust of research, and others as described by participants. The complete interview guide is provided in supplementary materials.

### Data analysis

Before analysis, investigators created preliminary themes based on the main interview questions on the following topics: (1) Challenges with SUD treatment and recovery, (2) Reasons for substance misuse, (3) Impact of substance misuse, and (4) Research priorities related to SUD treatment and recovery. This provided a structured framework for the analysis.

Analyses focused on site‐specific research priorities at the institutional or community level. Demographic data, including age, gender, and Tribal affiliation, were collected from participants. Researchers transcribed focus group recordings and analyzed post hoc, utilizing NVivo 14 analytic software for coding and thematic analysis. Initial coding of the qualitative data was conducted by the PI and research assistant using the NVivo Cloud Collaboration feature. This platform facilitated simultaneous coding and real‐time comparison of coded data, enabling the research team to establish consensus on emerging themes. Investigators then explored relationships among the categories and worked to meet consensus on the meaning of each code, theme, and category. Once finalized, the codebook draft was shared, and findings were presented to the research team, providing an opportunity for sharing and receiving feedback and gathering additional thoughts on the codebook content. In five iterative rounds of coding, new codes emerged inductively. They were continuously re‐evaluated, reshaped, and renamed to better align with the pre‐defined themes and context of the data.

## RESULTS

Forty‐seven individuals, 18 years or older, were recruited from seven sites and participated in the eight focus groups. The majority of participants identified as female (*n* = 32; 68.0%). Forty‐one participants identified as AI/AN (72.3%), 6.4% identified as White (*n* = 3), 2.1% identified as Native Hawaiian/Pacific Islander (*n* = 1), and 4.3% identified as Multi‐Racial (*n* = 2); 7 participants did not provide a racial identification (14.9%). Eighteen participants reported on ethnicity; of those, 16 (88.9%) identified themselves as non‐Hispanic. During project development, discussions with behavioral health directors led investigators to use snowball sampling to identify key stakeholders for participation in the study. Focus group participants included behavioral health directors, medical (i.e., physicians, nurses) and behavioral health (i.e., social workers, mental health counselors, substance use counselors) clinicians, Tribal leaders (i.e., Elders and elected members of Tribal Councils), individuals with lived experience, and Tribal community members from the Great Plains. Representative quotes from participants are presented in the supplementary materials.

**Figure 1:**
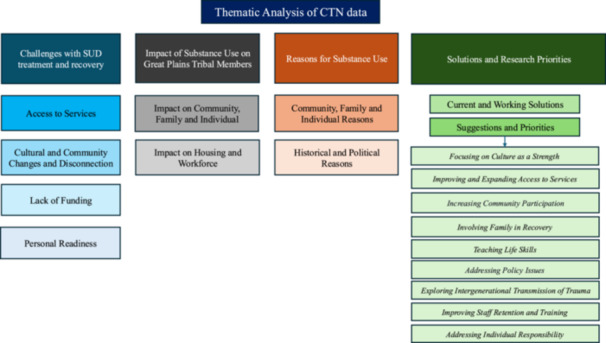
Thematic analysis of Aggregate Great Plains initiative data.

Through data analysis, investigators identified four themes: (1) challenges with SUD treatment and recovery, (2) impact of substance use on Great Plains tribal members, (3) reasons for substance use, and (4) solutions and research priorities. Theme four was the most prominent, with many statements relating to the importance of focusing on culture as a strength (see Figure 1).

### Theme 1: Challenges with SUD treatment and recovery

Participants discussed the challenges of dealing with SUD treatment and recovery throughout the interviews. These challenges are due to a variety of reasons, including community members' access to and funding for appropriate treatment services, an overall sense of cultural and community disconnection, and the readiness of those with SUDs to seek treatment and recovery.

Many respondents spoke about the lack of access to prevention, treatment, and recovery services. From provider shortages resulting in long wait times for treatment and counseling to a lack of culturally relevant and sensitive care, many community members are grappling with inadequate support.

Lack of aftercare, once treatment has been completed, was also seen as a major challenge for sustained sobriety. Without access to sober housing and other supportive services posttreatment, those in recovery are more likely to relapse when surrounded by others who may still be actively using substances. The respondents also called for longer treatment options, suggesting that 30‐day inpatient treatment is not sufficient for long‐term recovery and healing.

The funding challenges identified by participants greatly affected the effectiveness and sustainability of substance use treatment programs available for tribal members. These financial challenges ranged from the absence of adequate funding altogether to managing inconsistent funding streams, dealing with overly stringent controls, and administrative discourse over resource allocation.

Participants noted the challenge of reconnecting many community members with their traditional cultures. Elders, seen as vital community mentors, appear to be experiencing an increasing disconnect with others in the community, especially youth, impacting the passage of cultural knowledge and spiritual guidance. Respondents spoke of a loss of communal activities such as powwows, drumming, singing, and other gatherings that once provided bonding and education.

In some cases, despite the availability of treatment and recovery services, individuals may lack the personal readiness to seek help for substance use disorders and other mental health issues. The willingness to change is crucial in the recovery process and must be rooted in a commitment to benefit one′s family and larger community.

### Theme 2: Impact of substance use on Great Plains tribal members

Participants expressed deep concern about the profound impact of substance use on families, particularly children. The prevalence of neglect and the removal of children from their communities into nonnative households through the court system evoked a sense of loss and fear of cultural disconnection among respondents. Grandparents often assume caregiving roles in the absence of capable parents, and truancy rates are alarmingly high, which raises concerns about the long‐term impact of early school dropout. There was a shared desire to break the intergenerational cycle of trauma driving children towards substance misuse.

Respondents shared how substance use has led to increased crime, fear of victimization, vandalism, and anxiety over the trafficking of Native women and children. Prejudice and harmful stereotypes negatively impact the psychosocial well‐being of Native communities; for example, some respondents discussed frustration with county jails profiting from the arrest of Native people. Moreover, criminal background checks create barriers to employment and housing for those with substance‐related charges, which has resulted in a rise in homelessness. Many live in overcrowded conditions, making it difficult to maintain sobriety when others in the household are actively using substances. Lack of transportation further restricts access to employment, forcing some to return to harmful behaviors to survive the lack of stability, income, and opportunities.

### Theme 3: Reasons for substance use

Participants identified trauma as a primary driver of substance use, with sexual abuse, domestic violence, grief, and hopelessness leading individuals to use substances as coping mechanisms. Intergenerational trauma stemming from events throughout history, such as the massacres at Wounded Knee and other colonial practices that disrupted traditional ways of living and family dynamics still affects communities profoundly, perpetuating addiction.

Furthermore, participants noted that recovery from substance use can be difficult due to a shortage of 12‐step programs, delays in counseling services, and a lack of posttreatment support, leaving individuals to return to environments where substance use is prevalent and normalized. Participants highlighted how a lack of meaningful community activities and events to look forward to may lead to substance use due to diminished connection to others.

### Theme 4: Solutions & research priorities

#### Current and working solutions

While limited access to SUD treatment and recovery services is widely recognized as a significant barrier in the Great Plains region, several respondents also highlighted effective solutions that are currently available. Culturally relevant approaches, such as talking circles, *inipi* (sweat lodge ceremonies), and other traditional practices, play a crucial role in treating SUD. Participants highlighted the importance of culture and tradition in the acute stages of treatment and recovery, but also in prevention. Ongoing efforts, such as Lakota language instruction in public schools, are seen as a form of prevention, helping younger generations strengthen their cultural ties and promote resilience.

#### Focusing on culture as a strength

Many participants spoke about the resilience embedded in Native culture. Despite the challenges posed by substance use, participants recognized that the solutions lie in the inherent strength of individuals and tribal communities. However, a perspective and strategy shift is needed to tap this potential fully, specifically a move away from deficit‐based frameworks toward approaches that center Indigenous cultural strengths and self‐determined solutions. The participants stressed the importance of uplifting values such as hope and optimism in future research, which aligns with calls from Indigenous scholars to advance strengths‐based health policy and research that honors Indigenous leadership (O′Keefe et al., 2023; Redvers et al., 2023).

#### Improving and expanding access to services

Participants underscored the need for longer, more flexible treatment options for community members. When treatment durations are set and enforced by specific time frames, often based on the Western philosophy of substance use treatment, it can negatively impact the treatment process and outcome. For example, limiting a treatment stay to 30 days does not necessarily consider what is needed for someone′s ongoing care and support.

Participants also called for increased collaboration between treatment programs and other community services, such as supportive housing, to ensure continuity of care and adequately address the needs of people dealing with substance use and mental health issues. Additionally, they emphasized the need for more mental health counselors on staff at treatment centers to improve access to care.

#### Increasing community participation

Many respondents believed that increasing community connectedness is crucial in promoting healthier lifestyles and preventing dysfunction. Several respondents called for Tribal leadership to develop a strategic plan and act on building healthier communities that encourage sobriety and for Elders to have more significant roles in providing community healing.

#### Involving family in recovery

One of the research priorities suggested by participants was to compare the effectiveness of programs that involve families in recovery to programs that do not, acknowledging that family‐centered programs would better align with traditional Native values. Additionally, participants called for more community activities that would bring families together and promote cultural reconnection, such as celebrations for completing inpatient care for SUD.

#### Teaching life skills

An area of interest among respondents was addressing the needs of individuals transitioning out of SUD treatment centers or those experiencing homelessness. Participants advocated for initiatives that teach practical life skills, for example, household skills like cleaning and repairs, as well as entrepreneurial skills aimed at instilling hope and creating pathways to employment. Respondents expressed the value of cultural activities for youth, such as making *inipi* dresses, setting up teepees, and working with buffalo, which they saw as a way to strengthen youth cultural identity. Research by Usborne and Taylor (2010) demonstrated that culture‐based identity clarity is positively linked with self‐esteem and well‐being. According to behavioral health organizations involved in this study, there are adequate experiential data to show that cultural identity‐development addresses addiction, behavior health management, trauma, grief, abuse, and everyday life challenges encountered by American Indian people, and more of that is required for community‐level healing (Oaye Luta Okolakiciye, n.d.). The research agenda therefore, to build an evidence base supporting *Ceremony Assisted Treatment* of SUD, has been initiated through the University of North Dakota.

#### Addressing policy issues

Participants discussed how historical and present‐day government policies impact Native communities and emphasized the importance of returning to traditional values in addressing community challenges. They advocated for community‐centered solutions, such as increasing access to detox centers and crisis support services. Participants also proposed partnerships with institutions like Oglala Lakota College to expand local initiatives, establish a Native American Resource Center in Rapid City, improve data collection, and pay greater federal attention to the specific needs of Native communities.

#### Exploring intergenerational transmission of trauma

Participants discussed the importance of exploring the root causes of substance use in families to break the intergenerational cycle of trauma and dysfunction. One respondent noted that knowing the origins of trauma within families may help find targeted solutions to address them. Moreover, addressing the impact of SUD on maternal and child health requires increased community education on the effects of perinatal substance use. Increasing education in schools for young children on the benefits of a holistic lifestyle and the harms of substance use was recommended.

#### Addressing individual responsibility

Respondents highlighted the importance of individual responsibility in recovery from SUD to avoid developing a victim mentality despite historical and current adversities, instead of individuals in treatment programs blaming providers for their lack of success in recovery. Additionally, awareness campaigns on community resilience were recommended to promote a sense of strength and pride.

#### Improving staff retention and training

Respondents emphasized the importance of having trauma‐informed providers working in tribal communities. Nonnative providers may struggle to understand why healing can be a gradual process. Consistency and continuity in care were also highlighted; individuals seeking treatment are more likely to succeed when they have a relationship with their providers. To ensure continuity of care, it is essential to address staff turnover rates: participants recommended increasing the pay of mental health providers and examining what makes staff feel appreciated to address gaps in provider support.

## DISCUSSION

The following community‐driven research agenda (Table 1) was derived from the study data for Great Plains Tribal behavioral health services. This study agenda listing community priorities was developed from data from study participants. Many participants did not connect with the words “research priorities,” but responses linked to developing “solutions” provided robust ideas for further inquiry into addressing addiction and recovery. The process of qualitative analysis allowed for ranking priority issues and themes. The development of a codebook was a critical part of the deductive thematic analysis, which identified challenges, impacts, reasons, and solutions for substance use disorder. Results were presented to each participating site, and additional community engagement with these data resulted in research translation and follow‐up plans to operationalize community‐driven priorities.

**Table 1:** Community driven research agenda.

**1**	Examine how access to supportive housing can promote recovery from substance use.
**2**	Determine the efficacy of treatment and recovery programs that incorporate families compared to those that do not.
**3**	Explore the significance of the tiošpaye (extended family unit) in peoples' lives.
**4**	Examine how federal policies have shaped the lives of Tribal communities.
**5**	Collect data on the efficacy of cultural SUD treatment and recovery programs.
**6**	Explore the root causes of substance use in families.
**7**	Address the impact of substance use on maternal and child health.
**8**	Determine the individual′s responsibility in treatment and recovery, and how the burden of responsibility on providers be shared by the individual.
**9**	Research effective methods for recruiting and retaining staff.
**10**	Identify ways to show appreciation for providers.

The community‐driven research agenda described in Table 1 identifies 10 areas of opportunity for operationalizing research priorities. The core ideas are from community data, but language has been adapted to academic parlance that could be operationalized and translated into policy, programs, and resources. Agenda item #1 responds to the identification of a lack of sober housing as a challenge to recovery and supportive housing as a potential solution. Recommendations include policy research and advocacy to streamline criteria for drug and alcohol use for housing programs and building supportive housing infrastructure to support detox and recovery programs.

Item #2 addresses concerns raised regarding the impact of substance use on families and the need to involve family in recovery to align more effectively with traditional values. Recommendations for decolonized behavioral health programs serving AI patients include determining the efficacy of treatment that incorporates family participation compared to an individual focus. Incorporating the family in the recovery process can enhance behavioral health practice by creating home environments conducive to maintaining sobriety as a culturally aligned wise practice. Item #3 expands that circle of support in response to the identified need for community support to include all persons in their extended support system, or tiošpaye, whether part of their family of origin or close non‐relatives within their community. This approach aligns with findings from Skewes and Blume (2019), where a tribal community in Montana identified a multi‐level intervention system to address the individual, community, family, and systems levels.

Participants identified that both current and historical government policies have shaped Native communities and impact funding for recovery resources in Tribal communities. Agenda item #4 responds to that issue by providing an opportunity to more clearly define ways in which historical policies have contributed to the problem and changes to current policies that would benefit AI populations. Participants advocated for policy change at both the local and federal levels for investments in community infrastructure such as housing, transportation, and childcare in AI communities. Other recommended policy changes include increasing access to workforce education, increasing supportive housing opportunities for medium to long‐term recovery, and reimbursement of traditional healing for detox or behavioral health centers that choose to provide culturally based recovery services.

Item #5 responds to the identified need to provide culturally‐relevant elements of recovery. During data dissemination, participating behavioral health programs prioritized measuring outcomes that identify the healing impact of culture and ceremony on addiction recovery. Research supports this approach, noting that approaching the harmful behavioral health behaviors associated with acculturation requires structured opportunities for patients to reclaim traditional Indigenous wisdom and practices. This enables them to carry forward their cultural strengths to future generations (Warne, 2005). Despite a historically exploitive relationship with research institutions (Warne, 2006), the increase in Indigenous and culturally responsive researchers has started to rebuild the trust required to pursue the investigations detailed in the research agenda, especially with biospecimens or biological data (Mainous et al., 2023). Participants expressed the need to develop rigorous evidence on the efficacy of traditional healing. These programs want to host such studies to co‐generate credible and useful data for health providers and insurance payers. In response, one study researcher has begun a Ceremony Assisted Treatment study in collaboration with community members and traditional healers.

Participants identified trauma as a primary driver of substance use. The study highlights the need for trauma‐informed care training for healthcare professionals providing detox and recovery services, allowing behavioral health programs to address the impacts of colonization and the mental health repercussions of intergenerational trauma and loss. Items #6 and #7 provide an opportunity to explore the root causes of substance use in communities and to better understand and address the impact of SUD on maternal and child health to break intergenerational cycles of trauma and dysfunction.

Participants identified an opportunity to improve behavioral health services by emphasizing personal responsibility for recovery, reflected in Item #8. Focusing on individual responsibility and empowerment for recovery culturally aligns treatment and shares the ownership of healing between provider and patient (Warne, 2005). Incorporating cultural teachings in behavioral health programs moves clients through self‐development, on the spectrum from taking personal responsibility to family accountability and to community belonging (Two Dogs, 2024).

Item #9 responds to the community‐defined need to study effective staff recruitment and retention methods. Similarly, the National Indian Health Board published a comprehensive report on health workforce capacity in Indian country, citing additional need for staff and capacity for the Tribal behavioral health workforce (National Indian Health Board, 2024). These findings are consistent with previous research, which consistently identifies the availability of substance use disorder providers in AI/AN communities as a significant barrier (Herron & Venner, 2023). Expanding upon this theme, Item #10 addresses data from interviews and focus groups, expressing that innovative ways to show appreciation for providers would add value to behavioral health programs to boost staff retention through cultural values of appreciation and honoring of healers.

In accordance with TPR and other CBPR methods, disseminating research data back to participant communities closes the loop in creating community‐based knowledge (McDavitt et al., 2016). Data dissemination occurred through a series of community meetings following the release of the completed data report to the National Institutes on Drug Abuse. In tandem, the research team crafted a dissemination plan, considering the local context of each behavioral health program and its specific leadership and points of contact. The research team coordinated with each entity to present in person at standing meetings, a best practice in TPR/CBPR to avoid additional burden to community stakeholders (McDavitt et al., 2016), one‐to‐one sessions, lunch and learn staff gatherings, and in one case, an impromptu detox center staff presentation. Data were presented to each behavioral health program director and staff as presentation slides for their unique data, hard copies (printed) of the aggregated data report from all sites, including the Community Driven Research Agenda, and an individual data report.

Additionally, researchers provided electronic copies to behavioral health directors on a flash drive inside a gift bag with culturally appropriate gifts. Discussions from data dissemination meetings were rich and reflected the data presented thus far. Notes from these meetings were documented in a dissemination form that included space to allow for comments from the community regarding slide presentations, and actionable steps that behavioral health directors and staff members directly expressed. The next steps also included advocacy for policy and program development that can improve outcomes. The areas for policy advocacy are outlined in Table 2.

**Table 2:** Areas for policy advocacy.

Establish trauma‐informed care as a training requirement for detox/recovery providers. Behavioral health programs must directly address the impacts of colonization and the mental health repercussions of cultural genocide and loss. Reclaim cultural practices essential to the American Indian community′s healing. Behavioral health programs should adopt and implement policies that allow them to expand culturally appropriate care into existing service offerings. Increase policy advocacy for physical infrastructure (i.e., transportation, childcare, and the digital divide) to low‐income rural and Tribal communities. Expand workforce development resources for staff. Increase access to supportive housing for recovery. Increasing behavioral health coordination with systems influencing family dynamics, such as foster care, culture‐based parenting classes, and economic assistance.

### Limitations

Several limitations should be considered when interpreting the findings of this study. First, as a qualitative research study, the data are inherently context‐ and community‐specific and may not be transferable beyond the Great Plains Tribal communities involved in this study. While qualitative methods provide rich, in‐depth insights, they are subject to researcher interpretation and potential coder bias during the thematic analysis. To mitigate this, the analysis consisted of multiple rounds of coding by at least two coders and team consensus; however, a degree of subjective interpretation remains.

Second, sample size and representation present limitations. Lengthy and cumbersome federal approval processes limited our ability to engage with Indian Health Service (IHS) sites. As a result, we limited our engagement to only Tribal and urban AI programs. Although the study included 47 participants who have professional or community experience with various urban and reservation‐based substance use treatment programs or the community challenges caused by substance use, the findings may not fully capture the perspectives of all AI communities in the region. Additionally, the study's focus on behavioral health professionals and community partners may have led to an emphasis on systemic and service‐related challenges rather than the lived experiences of individuals directly experiencing substance use disorders. Staff turnover at several sites, which was frequently cited as a barrier to care, contributed to delays in the communication of study results.

Third, while purposive and snowball sampling helped identify key informants, this approach may have excluded individuals who are more difficult to reach, such as those not engaged in formal treatment services or those experiencing homelessness. This limits the ability to explore diverse perspectives on substance use and recovery fully. Furthermore, the reliance on self‐reported data in focus groups poses another limitation, as participants may have consciously or unconsciously responded in ways that align with community or research expectations.

Finally, demographic data such as age, gender, and race were collected from participants. However, providing this information was voluntary, and 7 of the 47 participants chose not to report their racial identity. Therefore, a statistical analysis of participant demographics was not performed to prevent inaccuracies. Despite these limitations, the study′s strengths lie in its culturally sensitive and participatory approach, and the in‐depth interviews provided valuable insights into the challenges and priorities of Tribal communities regarding substance use and recovery.

## CONCLUSION

The present study sheds light on the critical issue of substance misuse and the consequent community challenges in the Great Plains Tribal region. The study team gathered invaluable insights into the community‐defined needs and priorities for addressing these challenges through interviews and focus groups. While the findings highlight the barriers to accessing treatment, from policy issues to lack of funding, the most significant finding was the importance of integrating cultural strengths into treatment and recovery programs. Initiatives that emphasize Traditional healing practices and cultural values provide a more culturally grounded and relevant approach to addressing substance abuse in the communities. Additionally, addressing the root causes of addiction, along with early intervention and comprehensive counseling services, is essential for long‐term success.

Going forward, the Great Plains Initiative team will continue collaborating with community leaders to secure resources for exploring and addressing these priorities. The goal is to enhance treatment and recovery services to better meet community needs.

## CONFLICT OF INTEREST STATEMENT

The authors report no conflicts of interest. The submitted manuscript complies with APA ethical principles in the treatment of individuals participating in the described research. This study has been approved by the single Institutional Review Board of record at the University of North Dakota School of Medicine & Health Sciences (North Dakota).

## Supporting information

Supporting Information_supplement 1.

Supporting information_supplement 2.

supmat.
