# Supplementary material for: A community‐driven approach to address substance use and create a Great Plains American Indian addiction and recovery research agenda
Source: Am J Community Psychol. 2025 Dec 26;77(3-4):427–36. doi: 10.1002/ajcp.70039 (PMC12747506; doi:10.1002/ajcp.70039)
Supplement: Supplementary file 1 — Supporting Information_supplement 1. [file AJCP-77-427-s001.docx]

**Supplement 1: Focus Group Interview Guide Questions**

1. How does substance use and/or addiction impact the communities?
2. Which substances does the community consider most concerning, impact the communities most?
3. From your perspective, what challenges, costs, or complications has substance use or addiction caused in your communities?
4. Which factors, historical or contemporary, contribute to substance use, misuse, and addiction at the individual level?
5. Which of these factors, historical or contemporary, contribute to substance use, misuse, or addiction at the family level, in the home?
6. Which of these factors, historical or contemporary, contribute to substance use, misuse, and addiction at the community-level?
7. How does the history of these communities, related Federal-Indian policies, e.g., forced acculturation/assimilation, land dispossession, loss of culture/lifeways, and cultural discontinuity impact the problem, if at all?
8. How does the traditional cultural and spiritual ways of the Tribal community guide the relationship with the substances of use/misuse that we’ve discussed?
   1. How does this relationship differ for those community members or families that do not identify as traditional in spirituality, or cultural identity?
9. What solutions does this community offer those that have difficulty with substance use?
   1. Which solutions are community-based, traditional, cultural, or spiritual, and how are they different from Western medical solutions to addiction and recovery?
   2. What solutions are most effective, impactful, or best practices by the community?
10. How do these community derived solutions differ, if at all, from services that have been provided in the past?
11. Which community-based, local, state, or federal agencies and resources are important for healing, recovery and attaining wellness from substance use and addiction in your community?
12. What resources are necessary in implementing community-grounded solutions to addiction and recovery that we’ve discussed?
13. With all that we’ve discussed today, which components of a successful treatment program are most important for healing, recovery and attaining wellness from substance use and addiction in your community?
