# Supplementary material for: A community‐driven approach to address substance use and create a Great Plains American Indian addiction and recovery research agenda
Source: Am J Community Psychol. 2025 Dec 26;77(3-4):427–36. doi: 10.1002/ajcp.70039 (PMC12747506; doi:10.1002/ajcp.70039)
Supplement: Supplementary file 2 — Supporting information_supplement 2. [file AJCP-77-427-s002.docx]

**Supplement 2. Themes and Sub-Themes: Representative quotes**

| **THEME: CHALLENGES WITH SUD TREATMENT AND RECOVERY** | |
| --- | --- |
| **Access to Services** | *“And then there’s a lack of treatment centers and inpatient treatment centers. And then at that, it’s more long-term treatment centers are far and wide. The majority of them are like 30 days and then, (pause), that’s it. Then they come back and then they’re back in the same environment, so.”* |
| **Cultural and Community Changes and Disconnection** | *“And there's such fragmented cultural knowledge on our reservation that I think that that really impacts people's ability to be able to get help for their addictions.”* |
| **Lack of Funding** | *“When we do cultural activities, cultural events, people show up - the young people start to get engaged. So, I think it's important - we don't have the money or the time to spend on prevention that we'd like to.”* |
| **Personal Readiness** | *“But I don't hear a lot about how important a client's responsibility is in all of this. More so, because we're just there to help. But I don't see a lot of focus on that. What's the client's responsibility? And not all of that is put on our shoulders”.* |
| **THEME: IMPACT OF SUBSTANCE USE ON GREAT PLAINS TRIBAL MEMBERS** | |
| **Impact on Community, Family and Individual** | *“I really don't know of a level where substance abuse does not impact our community. So, and I don't know if it's in order of um, of the severity of the impact. But we're talking our families. We're talking employment. We're talking incarceration. We're talking birth defects. We're talking spirituality. I mean I. I just don't know of a realm where addiction does not impact our community. I really honestly cannot think of one. Being totally honest.”* |
| **Impact on Housing and Workforce** | *“Meth use really affects housing around here. If you drive to the communities, there's boarded up houses everywhere.” “You know, they don't have transportation. So, if they don't have transportation, they don't have employment opportunities. So, you know how they going to survive and go back to doing what it was they were doing because they ain’t going to starve, you know.”* |
| **THEME: REASONS FOR SUBSTANCE USE** | |
| **Community, Family, and Individual Reasons** | *“The community doesn't have anything to look forward to. There's no kind of community centers or anything. And you know, if they want to really get involved, they need to have money to participate and all that. ... It gets overwhelming sometimes.”* |
| **Historical and Political Reasons** | *“The government process of eliminating us for killing us and punishing us is still in effect today, this is why we're overwhelmed with the trauma. The intergenerational trauma, the historical trauma carrying, or genetically so where we can some ... of the situations we come upon, we can't handle it. So, we're getting all, we medicate with this. And what do we? And we don't even know that, you know, we're medicating all these overwhelming feelings. So, it's ... getting worse and worse...”* |
| **THEME: SOLUTIONS & RESEARCH PRIORITIES** | |
| **Focusing on Culture as a Strength** | *“We take our participants at every solstice, every equinox, we take them to... our parts of our... Black Hills here; this is our homeland, our spiritual homeland. This is where our creation story comes from. So we take them to these different sites and explain why we've been doing this for thousands and thousands of years. And it's really not only for the youth, the adults, too, you know, because this is the first time they're hearing it, too. So it really brings back that brings upon that like I said, self-esteem, confidence, education to the that. We have a way to, and then they start to understand a lot of things that's going on in their life.”* |
| **Improving and Expanding Access to Services** | *“And I know that the... recreation center, which addresses homelessness, they’ve started, homelessness or houselessness, they’ve started AA meetings five days a week, you know, but that’s just – those are just really fragmented piece, little piece services, and it would be nice to have a whole system for mental health disorders and substance abuse being the probably the biggest majority of those disorders.”* |
| **Increasing Community Participation** | “*My grandpa has said the essence of a Traditional leader is not somebody who has all these titles or things that we see in modern society or who’s the richest. It’s the person who seen what was needed to be done for the people and took the initiative to get that done.*” |
| **Involving Family in Recovery** | *“I know when we... increase the interaction with the families, there is a lot more interaction [with] the people that need our services because they got more support. When they're standing alone, they pretty much isolate. They don't reach out. But when we involve the family, they start reaching out to their friends, family and become part of the tiošpaye where everybody's involved. Because, you know, we had everybody involved in that recovery person's life and opening his chances of recovery are that much greater than if he stands alone. And we know that from our cultural values.”* |
| **Teaching Life Skills** | *“Like the schools when I was going to school, they used to have home economics and auto body shop and woodworking. Getting the kids more activated into stuff like that, teaching our young adults how to cook, how to even take care of a home. Cause a lot of them, I mean some of the houses that we go into, they're so destroyed by them not even knowing how to pick up a broom and sweep and mop. I mean I think we really need to educate our people that way too. Self-pride, self-care, self...just self-care basically.”* |
| **Addressing Policy Issues** | *“So, we got to step up and approach the state, the legislation, the nation, the world that...I guess we don’t have to, but we’re doing what we’re doing here and now it shows. But the funding part is.. How can we let America know that we’re who we are, you know, and that this way is effective, you know?”* |
| **Exploring Intergenerational Transmission of Trauma** | *“If we know what's causing it, then maybe we could help them try to fix what the trauma is for them. So if it's maybe if they saw, maybe they were physically abused while they were a kid, well, then we could work with them and help them deal with, ok, so number one, this problem that you're having, it didn't have anything to do with you. The issue was with your parent or the person who was physically abusing you. That's where the issue lies. So if we could help them to deal with that part of it, then maybe they wouldn't need to turn to alcohol or drugs.”* |
| **Addressing Individual Responsibility** | *“I believe that we're survivors and a lot of people, it becomes the norm after a while. And so, you know, like awareness campaigns, you know, like remembering who we are and what our people did to be resilient and survive and not have that mindset of like, “Oh my gosh, I'm a victim. And you know, this happened to me. I'm going to drink forever. And you know, I'm sorry, but this is our way of life now.’.”* |
| ***Improving Staff Retention and Training*** | *“I have a firm belief that if your employees feel appreciated and they feel like the work that they're doing is meaningful, they will do it for less. I don't think money's ever going to solve our problem. But when you talk about the scrutiny that we get. I mean, what's the average burnout rate of a clinician anymore, six years? How many doctors do you know that quit after six years? They don't! I can't emphasize enough if you want to do some research look at what provider, how providers feel that they're treated, and if they're appreciated, I'll promise you the money won't make the difference.”* |
