# Supplementary material for: A community‐driven approach to address substance use and create a Great Plains American Indian addiction and recovery research agenda
Source: Am J Community Psychol. 2025 Dec 26;77(3-4):427–36. doi: 10.1002/ajcp.70039 (PMC12747506; doi:10.1002/ajcp.70039)
Supplement: Supplementary file 3 — supmat. [file AJCP-77-427-s003.docx]

Figurementary Figure 1. Recurrence patterns

PCB, paclitaxel-coated balloon; PES, paclitaxel-eluting stent.

Figurementary Figure 2. The rate of CD-TLR in the patients with PP loss

PCB, paclitaxel-coated balloon; PES, paclitaxel-eluting stent; CD-TLR, clinically driven target lesion revascularization.
